# An intervention program with the aim to improve and maintain work productivity for workers with rheumatoid arthritis: design of a randomized controlled trial and cost-effectiveness study

**DOI:** 10.1186/1471-2458-12-496

**Published:** 2012-07-02

**Authors:** Myrthe van Vilsteren, Cécile RL Boot, Romy Steenbeek, Dirkjan van Schaardenburg, Alexandre E Voskuyl, Johannes R Anema

**Affiliations:** 1 Department of Public and Occupational Health, EMGO Institute for Health and Care Research, VU University Medical Center, Amsterdam, the Netherlands; 2 Body@Work, Research Center Physical Activity, TNO-VU University Medical Center, Hoofddorp, the Netherlands; 3 Jan van Breemen Research Institute | Reade, Amsterdam, the Netherlands; 4 TNO Work and Health, Amsterdam, the Netherlands; 5 Department of Rheumatology, VU University Medical Center, Amsterdam, the Netherlands; 6 Research Center for Insurance Medicine AMC-UMCG-UWV-VU University Medical Center, Amsterdam, the Netherlands

## Abstract

**Background:**

Workers with rheumatoid arthritis (RA) often experience restrictions in functioning at work and participation in employment. Strategies to maintain work productivity exist, but these interventions do not involve the actual workplace. Therefore the aim of this study is to investigate the (cost)effectiveness of an intervention program at the workplace on work productivity for workers with RA.

**Methods/design:**

This study is a randomized controlled trial (RCT) in specialized rheumatology treatment centers in or near Amsterdam, the Netherlands. Randomisation to either the control or the intervention group is performed at patient level. Both groups will receive care as usual by the rheumatologist, and patients in the intervention group will also take part in the intervention program. The intervention program consists of two components; integrated care, including a participatory workplace intervention. Integrated care involves a clinical occupational physician, who will act as care manager, to coordinate the care. The care manager has an intermediate role between clinical and occupational care. The participatory workplace intervention will be guided by an occupational therapist, and involves problem solving by the patient and the patients’ supervisor. The aim of the workplace intervention is to achieve consensus between patient and supervisor concerning feasible solutions for the obstacles for functioning at work. Data collection will take place at baseline and after 6 and 12 months by means of a questionnaire. The primary outcome measure is work productivity, measured by hours lost from work due to presenteeism. Secondary outcome measures include sick leave, quality of life, pain and fatigue. Cost-effectiveness of the intervention program will be evaluated from the societal perspective.

**Discussion:**

Usual care of primary and outpatient health services is not aimed at improving work productivity. Therefore it is desirable to develop interventions aimed at improving functioning at work. If the intervention program will be (cost)effective, substantial improvements in work productivity might be obtained among workers with RA at lower costs. Results are expected in 2015.

**Trial registration number:**

NTR2886

## Background

Rheumatoid arthritis (RA) is an autoimmune disease characterized by chronic inflammation of the joints [1]. Fatigue, stiffness and pain are prominent symptoms. RA has a fluctuating and chronic progressive course and may result in loss of function of the joints [2].

In northern Europe, the prevalence of RA is estimated at 6.6 per 1000 [3]. In the Netherlands, the prevalence was estimated at 7 per 1000 in men, and 11 per 1000 in women in 2007 [4]. The work status of RA patients affects the costs for society for RA [5]. In a review of Lundkvist et al. (2008), the economic impact of RA on society was reviewed. Overall, the mean annual costs per patient were estimated at around 13500 Euros in Europe. Besides medical costs and drugs, which represent for about one-third of these costs, productivity losses account for a substantial part of the total costs of RA (32%). Other costs include informal care costs, and non-medical costs such as formal home help [3].

**Figure 1:**
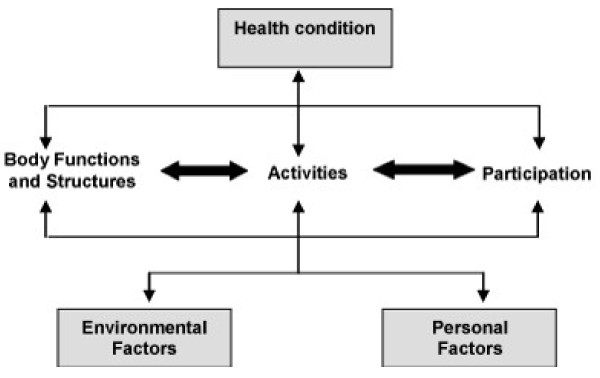
**International classification of functioning, disability and health **[6]**.**

The International Classification of Functioning, Disability and Health (ICF, Figure 1) describes participation as all aspects concerning activities (execution of a task or action) and participation in daily life (involvement in a life situation) [6]. RA patients often experience restrictions in participation in employment. A Dutch cohort of RA patients showed that RA patients have higher work disability rates when compared to the Dutch population; after a mean disease duration of 4.3 years, RA patients had a 2.14 risk of being work disabled [7]. In another Dutch cohort of patients with early arthritis (EAC), 19% of the patients became work disabled over a period one year before and one year after entering the EAC [8]. In a review study, it was reported that 66% of employees with RA reported work loss due to RA in the past year, with a median duration of 39 days [9]. RA patients reported to make adaptations in their work environment in an attempt to continue working, but were still afraid of losing their job [10].

The health care available for Dutch RA patients is strictly separated into an occupational health system and a curative health system. Communication between medical specialists and occupational physicians/therapists is poor and the patient often has to deal with conflicting advises [11]. Consultations with the rheumatologist do not concern work and work-related problems the patient might be facing and furthermore, usual care does not include consultation with the occupational physician; patients only visit the occupational physician in case of sick leave [12]. The current health care for RA patients is mainly focused on treating the health condition with little attention for environmental and personal factors, while, in light of the ICF model, attention for environmental and personal factors is crucial in improving participation.

The Dutch Health Council stated that there is a strong need for multidisciplinary recommendations for (resumption of) work activities in chronic diseases [13]. Previous research on the effect of integrated care programs on work participation in chronically ill patients has shown that integrated care might increase work participation [14,15]. In general, suboptimal care by lack of communication may lead to long-term absenteeism from work and permanent disability, which are associated with high costs [13,16].

Because of the need for recommendations for work activities in chronic diseases and the fact that involving external factors (i.e. the worksite) might make a difference, this study aims to develop and implement a structured, well-coordinated intervention program intended to coordinate the clinical care and occupational care at the worksite and thereby support work participation of workers with RA. Integrating clinical and occupational care will lead to one treatment goal for the involved caregivers, namely improving work productivity. Hereby, we aim to form one treatment advise for the patient, so the patient does not have to deal with conflicting advises. In contrast to previous studies, the current intervention will be executed at the worksite, involving the patients’ supervisor and the primary aim is to maintain and increase work productivity instead of health outcomes. The workplace intervention, which is based on participatory ergonomics, will be tailored to the individual worker, making it possible to address issues such as workload, decision latitude, communication between employee and supervisor, and reducing barriers at the workplace. Furthermore, the current study focuses on improving and maintaining work productivity, instead of job loss.

The aim of this paper is to describe the design of a randomized controlled trial (RCT) comparing the intervention program with usual care for patients with RA. The first objective of this study is to evaluate the (cost)effectiveness of the intervention program on work productivity, measured by hours lost from work due to presenteeism. The second objective of this study is to evaluate the process of implementation (experiences, acceptance, barriers, and facilitators) of the intervention program.

## Methods

### Organization of the study

The design of this study is an RCT with a follow up of one year. Patients will be recruited from Reade (a specialized rheumatology center, formerly the Jan van Breemen Institute), Amsterdam, the outposts of Reade, and the department of rheumatology of the VU University Medical Center, Amsterdam, the Netherlands.

The Medical Ethics Committee of the Slotervaart Hospital and Reade, and the VU University Medical Center, Amsterdam, the Netherlands approved the study design, protocols, procedures, and informed consent. Participation is voluntary and all participants will sign informed consent. Towards the involved stakeholders (employees, supervisors and all involved caregivers) the study is entitled the “Care for Work” study.

### Participants

The population consists of RA patients (18–64 years) who have visited a rheumatologist of one of the participating hospitals during the last year. Eligible patients are diagnosed with RA, and have a paid job (paid-employment or self-employed) for at least 8 hours per week. Furthermore, eligible patients experience difficulties in functioning at work. Patients will be excluded from the study in case of severe comorbidity that will hamper compliance to the protocol, when unable to read or understand Dutch language, and when taking more than 3 months sick leave at time of inclusion.

Eligible patients will receive an information letter about the project from their own rheumatologist three weeks before the consult with the rheumatologist. This letter includes a reply card which patients can use to point out whether they want to participate in the study or not. Patients who return the reply card and indicate to be willing to participate in the study are contacted by the researcher by telephone. In this contact, the researcher provides additional information about the implications of participation and checks the eligibility of the patient based on the inclusion and exclusion criteria. If a patient meets all selection criteria, the researcher plans a face-to-face appointment with the patient, preferably immediately before or after the consultation with the rheumatologist. When this is not possible, the face-to-face appointment is planned on a different date. In addition, patients are offered the opportunity of a home visit. During the face-to-face appointment, the patient will be asked to sign informed consent, and complete the baseline questionnaire. Furthermore, randomisation will be performed.

### Randomisation

Randomisation to either the intervention group or control group will be performed on patient level. To prevent unequal randomisation, patients will be pre-stratified by 3 prognostic factors; sex, whether the patient performs heavy physically/mentally demanding work or light physically/mentally demanding work, and the number of working hours per week. To determine whether a patient performs physically/mentally heavy or light work, a classification of De Zwart (1997) will be used, which is based on the subdivision of all occupational classes in the Netherlands [17]. The third stratification factor is the number of working hours per week. Work hours will be divided into high and low; high represents 20 work hours per week or more, and low represents less than 20 work hours. Randomisation will be performed using minimisation [18]. Minimisation was chosen because this method makes it possible to balance groups for several prognostic factors, even in small samples [18,19]. Minimisation aims to ensure treatment arms are balanced with respect to predefined patient factors as well as for the number of patients in each group. Minim is a software program based on the minimisation method, which will be used for treatment allocation [20]. The result of the randomisation will be kept in a sealed envelope before handing the envelope to the participant.

### Interventions

All participants will receive usual rheumatologist-led care. The participants in the intervention group will also take part in the intervention program. The intervention program consists of two components which complement each other; integrated care including a participatory workplace intervention. Both are described below.

#### Intervention component 1: Integrated care

Integrated care will be provided based on a case management protocol which will be executed by a multidisciplinary team. This team consists of a trained clinical occupational physician (who will act as care manager), a trained occupational therapist, and the patients’ own rheumatologist and occupational physician.

The care manager has an intermediate role between clinical and occupational care. He is responsible for the planning and coordination of care, and for communication between all members of the multidisciplinary team, the patient’s supervisor and general practitioner.

The patient will visit the care manager within one week after randomisation. The care manager starts with history taking and physical examination. History taking aims to identify functional limitations at work and factors that could influence functioning at work. By the end of the first consultation, the care manager proposes a treatment plan. If the patient agrees with the plan, the care manager will contact the patients’ rheumatologist in order to advise the plan for adjustments at work. When the patients’ rheumatologist agrees with the plan, the care manager will send the treatment plan to the other members of the multidisciplinary team. If the rheumatologist does not agree with the treatment plan, the care manager will have contact with the rheumatologist, discuss the treatment plan and achieve consensus.

**Figure 2:**
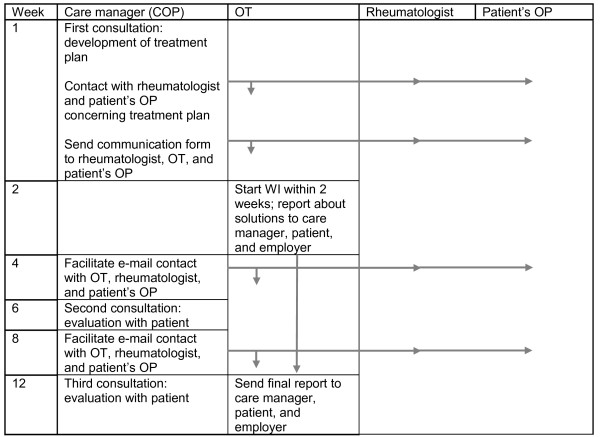
**Time scheduling of the multidisciplinary intervention program.** COP = clinical occupational physician; OT = occupational therapist; OP = occupational physician; WI = workplace intervention.

Another task of the care manager is to coordinate communication between the members of the multidisciplinary team. This will occur during a conference call with all members, or by sending coded emails to all members. The patient will visit the care manager again after 6 and 12 weeks to evaluate and if necessary adjust the treatment plan. Figure 2 depicts the flow of the intervention program.

#### Intervention component 2: Participatory workplace intervention

The workplace intervention concerns workplace adaptations and is based on active participation and strong commitment of both the patient and supervisor. The workplace intervention is based on methods used in participatory ergonomics [12,21,22]. The workplace intervention will be coordinated by the occupational therapist, and executed by the patient, the patients’ supervisor, and other potential stakeholders (e.g. a human resource manager). The aim of the workplace intervention is to achieve consensus between patient and supervisor concerning feasible solutions for the obstacles for functioning at work. After consensus, the occupational therapist, patient, and supervisor agree on a plan of action. Responsibility for implementing the plan of action is put on the patient and the patients’ supervisor’s account as much as possible. For this intervention, two occupational therapists will be trained by an expert to coordinate the workplace intervention. Table 1 describes the steps of the workplace intervention.

**Table 1:** Steps of the workplace intervention protocol

**Step**	**Workplace intervention protocol**
1	Within 2 weeks after the patient visits the care manager, the OT makes an appointment by telephone with the patient and the patient’s supervisor for the first visit of the workplace intervention protocol.
2	First visit consists of: 1. Patient’s workplace observation and inventory and ranking of patient’s tasks and obstacles for functioning at work by the patient. 2. Inventory and ranking patient’s tasks and obstacles for functioning at work by the patient’s supervisor. 3. Patient, patient’s supervisor and OT brainstorm and discuss solutions to clear the obstacles for functioning at work.
3	Within two days after the OT has visited the workplace, the OT reports about all solutions and actions in a report to the patient, the patient’s supervisor, and the care manager.
4	An optional worksite visit to give additional instructions or training to the patient will take place if necessary. The moment of instruction depends on whether adjustments on the worksite have to be made first.
5	Four weeks after the first visit, an evaluation will take place by telephone between the patient and OT concerning the implementation of solutions agreed upon. If necessary, a stakeholder has to be found for further support of improvements.
6	Within two days after the telephone evaluation, a final report is sent to the care manager to report the progress of the protocol.

OT, occupational therapist.

### Outcome assessment and data collection

#### Outcome measures

##### Primary outcome

The primary outcome of this study is productivity, measured by hours lost from work due to presenteeism. Presenteeism is the problem of being on the job but, because of medical conditions, not being able to fully function. Presenteeism will be measured by means of the Work Limitations Questionnaire (WLQ) [23]. Based on 25 items, a score will be calculated which presents the percentage of productivity loss. This score will be multiplied by the number of work hours per week, leading to an estimation of the hours that a patient was not fully productive. The WLQ consists of four dimensions; physical demands (Cronbach’s alpha 0.93), time management (Cronbach’s alpha 0.95), mental-interpersonal demands (Cronbach’s alpha 0.97), and output demands (Cronbach’s alpha 0.96) [24]. Presenteeism will be measured at baseline, and after 6 and 12 months. The recall period of the WLQ is 2 weeks. The validity and reliability of the WLQ concerning RA have been shown in several publications [24-26].

##### Secondary outcomes

Sick leave will be measured with modules C and D of the Productivity and Disease Questionnaire (Prodisq) [27]. The Prodisq is developed based on the Quantity and Quality (QQ) method and provides a reliable and valid tool for measuring quantity and quality of work on a daily basis [28].

Quality of life will be measured by means of the RAND-36 and EQ-5D-5L [29,30]. The RAND-36 consists of 8 subscales, namely physical functioning, role limitations due to physical problems, social functioning, bodily pain, general mental health, role limitations due to emotional problems, vitality, and general health perceptions [31]. The EQ-5D-5L is an improved version of the Euroqol (EQ-5D). In contrast to the Euroqol, the EQ-5D-5L has 5 levels of severity in 5 dimensions, in stead of 3 levels of severity in 5 dimensions, which improves the instrument’s sensitivity [30]. The Euroqol has been validated in a population of RA patients [32].

Pain and fatigue will be measured with single items using visual analogue scales (VAS) [33,34]. Studies have shown that a single item VAS for fatigue and pain performs as well as or better than longer scales in respect to sensitivity to change [34,35].

The subscale ‘supervisor social support’ of the Job Content Questionnaire (JCQ) will be measured as effect [36].

The RA Work Instability Scale (RA-WIS) will be used to measure work instability [37,38]. The RA-WIS consists of 23 statements which are answered by ‘yes’ or ‘no’. The RA-WIS can be scored in three bands by counting the number of items answered by yes. A score less than 10 indicates low work instability, indicative of low risk of work disability. Scores in the range of 10–17 indicate moderate work instability. Scores above 17 are high scores of work instability and these individuals can be considered at high risk of work disability.

##### Prognostic measures

All prognostic measures will be collected at baseline. Sociodemographic data will be collected such as age, sex, education, working hours per week, and job description. Medication used will be collected from the patient records. Furthermore, daily functioning will be measured at baseline, by means of the Health Assessment Questionnaire (HAQ) which is a reliable, valid questionnaire widely used in RA research [39]. In addition the Disease Activity Score (DAS28) will be included as a prognostic factor. The DAS28 is assessed as a part of usual care by the rheumatologist and will be obtained from the patient records.

Psychosocial job characteristics will be examined by using the JCQ. The following subscales will be used as prognostic measures; Decision authority; Psychological job demands; Physical job demands; Co-worker social support [36].

##### Process evaluation

The process evaluation aims to examine experiences with the intervention program. The process evaluation will be carried out using both quantitative and qualitative techniques; a short questionnaire will be composed and interviews will be conducted after 6 months follow up. Data will be collected from the participants, the participants’ supervisors, and the health professionals involved (clinical occupational physician, occupational therapist and rheumatologist).

Process measures for the process evaluation include compliance to the intervention, realisation of the advices and adjustments at work, and satisfaction with the intervention. We will focus on the reach (part of the target group that participates), dose delivered (efforts of the health professionals), dose received (extent to which the patient has put effort in the intervention), and fidelity (extent to which the intervention was delivered according to the protocol). The process measures that will be examined in the process evaluation will be based on the model of Linnan and Steckler (2002) [40].

##### Cost-effectiveness evaluation

Cost-effectiveness of the intervention will be evaluated from the societal perspective whereby the costs and benefits will be captured independently of those who bear the costs and those who receive the benefits. Health care costs, productivity costs and costs in other sectors will be measured and valued. Health care costs comprise costs directly related to the provision of health care, such as costs for primary and secondary care, but also costs for drugs and alternative treatments. The health care costs will be calculated by using tariffs for the costs of health care professionals based on the cost calculation guidelines for health care in the Netherlands [41]. The prices of prescribed drugs will be based on Daily Defined Dosage (DDD) taken from the Royal Dutch Society for Pharmacy [41]. The direct non-health care costs are calculated by using the information obtained from the cost questionnaires and shadow prices. The costs directly related to the development and implementation of the intervention will be registered. Productivity costs are not related to health care, but are costs in paid labour as a consequence of sickness, sick leave, disability of a productive person. The most important productivity costs will be measured in terms of lost productivity: presenteeism, absenteeism and compensation mechanisms. Lost productivity will be calculated by using the Friction costs method which basically multiplies the days of production loss till replacement by the average day wage [41]. For the latter method, the Dutch guideline for economic evaluation is used [41].

Detailed information concerning the methods for economic evaluation can be found in the article by Noben (2010) [42].

##### Sample size

The sample size calculation is based on the primary outcome of this study, which is productivity at work, measured by hours lost from work through presenteeism, measured by the Work Limitations Questionnaire (WLQ). We assume that a difference in the score of two hours lost per two weeks due to presenteeism is a relevant difference. This is based on a recent study, where an average number of four lost hours per two weeks (SD: 3.9) was measured among patients with RA by using the WLQ [43]. A two hour per two weeks difference implies a moderate standardized effect of 0.5. Power analysis revealed a sample size of 71 participants per group, assuming a dropout rate of 15%. This implies that 142 patients will have to be included in total. The difference in score of the WLQ of two can be detected with a power (1-beta) of 0.80 and an alpha of 0.05.

##### Blinding

Patients, therapists and researchers cannot be blinded for the allocated treatment after randomisation. Treatment allocation takes place after the informed consent form is signed, and the baseline questionnaire is filled out. It is not likely that the researcher or care providers will influence the way participants fill out the follow up questionnaires, since these will be sent either by mail or by email. All participants will receive their own personal code, based on the treatment center they attend and a run-up number. The research assistant will put all data in the computer by the personal code. Therefore, the analysis of the data by the researcher will be blind.

##### Co-interventions and compliance

The use of co-interventions cannot always be avoided during the intervention period. Asking patients and care givers independently about all interventions applied will assess the compliance to the intervention program evaluated in this study. Information about all treatments and co-interventions received by patients in both the intervention as well as the control group will be collected by means of questionnaires at 6 and 12 months follow-up.

#### Statistical analysis

##### Effect evaluation

The outcomes of the questionnaires will be compared between both groups at baseline and at follow-up. All analyses will be performed at patient level according to the intention to treat principle.

First, to examine the success of randomization, prognostic factors will be compared between the two groups by Student T tests and Chi square tests. In case of unsuccessful randomization, it will be checked whether the prognostic factor acts as a confounder or effect modifier.

The primary independent variable in the analyses will be the treatment group: intervention or usual care). The primary dependent variable is work productivity. Two analyses will be performed: a) linear mixed model with the outcome variable measured at follow-up as the dependent variable, adjusted for the outcome measured at baseline, and b) an analysis as above but adjusted for potential covariates (e.g. age, sex, type of work, time from diagnosis RA).

Effects of the intervention will be checked for effect modification (e.g. sex, HAQ score, time from diagnosis RA). All analyses will be performed with SPSS 18.0 (SPSS Inc. Chicago, Illinois, USA). Outcome variables will be assessed at a significance level of 0.05.

In order to assess whether protocol deviations have caused bias, the results of the intention-to-treat analyses will be compared to per-protocol analyses. Only those patients who complied fully with the intervention protocol will be included for the per-protocol analyses.

##### Cost-effectiveness evaluation

Bootstrapping will be used for comparison of mean direct, indirect and total costs between the two groups. Confidence intervals will be obtained to estimate the uncertainty surrounding these costs differences. Incremental cost-effectiveness ratios (ICER) are calculated by dividing the difference between the mean costs of the intervention by the difference between the mean effects of the intervention to indicate whether the additional costs needed for the intervention gain at least one extra unit of effect compared to usual care. The bootstrapped cost-effect pairs are graphically presented in a cost-effectiveness plane. The calculated ICER is compared with a threshold on the graph which indicates the maximum amount society is willing to pay for the intervention/outcome measure. Acceptability curves are calculated in order to show the probability of the intervention being cost-effective at a specific ceiling ratio. Furthermore, sensitivity analyses will be performed to process the robustness of the economic evaluation by examining changes in results when key variables are varied over a specific range (change cost prices, or calculation methods for (in)direct costs).

## Discussion

In this study, the cost effectiveness of an integrated care program including a participatory workplace intervention on work productivity for workers with RA will be investigated. In contrast to previous initiatives, the current intervention will be executed in a clinical and occupational setting. By integrating both aspects of health care, one treatment goal will be formed, namely improving work productivity. Furthermore, the workplace intervention will be tailored to the specific needs of a worker, making it possible to address difficulties the worker is experiencing while at work.

### Comparison with other studies

Work disability rates are higher in patients with RA as compared to the general Dutch population [7]. The work status of patients with RA affects the costs for society for RA [5]. As described before, Lundkvist (2008) shows that productivity losses account for one third of the total costs of RA [3]. These data highlight the need for interventions aimed to improve and maintain work productivity and hence, prevent job loss.

In 2002, a systematic review was performed to describe the effectiveness of vocational rehabilitation programs for patients with chronic rheumatic diseases [44]. This review identified six studies, all uncontrolled. Five of six vocational rehabilitation programs showed a marked positive effect on work status, but evidence for the benefit of these interventions is limited, due to methodological shortcomings.

A recent RCT in the Netherlands compared a multidisciplinary job-retention vocational rehabilitation program with usual outpatient care [45,46]. The multidisciplinary vocational rehabilitation programme aimed to guide patients and adapt an intervention to the specific needs of the patient. The basic assessment of the patient was performed by a rheumatologist. No differences were found between the two groups regarding the proportion of patients having lost their job at any time point.

The study of Allaire (2003) showed a significant delay of job loss in the intervention group compared to the control group [47]. The intervention consisted of three components; job accommodation, vocational counselling and guidance, and education and self-advocacy. The intervention was delivered by a rehabilitation counsellor employed by the study.

These examples show the need for intervention programs aimed to improve work productivity and prevent job loss, but up till now, no effective intervention programs on work productivity have been identified. What we aim to do differently in the current study is that the worksite and the patient’s supervisor are involved in the intervention, because previous initiatives that did not involve the worksite were not effective. The current intervention is based on the interventions evaluated in the studies of Lambeek (2010) and van Oostrom (2010) [14,48]. Both studies showed a positive effect of the workplace intervention.

#### Methodological considerations

A weakness of this study might be that all data is self reported. No objective data will be collected concerning sick leave, and objective data concerning work productivity is not available. There is still discussion about whether self reported data is reliable. However, several recent studies report that similarity between self reported data and data from for example a national insurance authority is high, and that self reported data is justified in research [49,50]. Despite the accuracy of self reported data, there is still a chance of recall or information bias. Because this study is a randomized controlled trial, we expect recall bias to occur to some extent in both treatment arms. Therefore, recall bias will not lead to an overestimation of study results. Besides this, we investigate differences in time for our primary outcome. It is likely that an over- or underestimation will be the same on all three time points; therefore, delta will not change. Our primary outcome, work productivity, is measured by a valid measurement tool [24]. The WLQ has been validated among patient with osteoarthritis.

The internal validity of this RCT might be affected by the fact that due to our study design and the character of the workplace intervention, blinding of patients and health care providers is not possible. A potential source of bias is the difference in attention patients receive, also called the Hawthorne effect [51]. The Hawthorne effect refers to the methodological effect in field experiments that patient’s knowledge that they take part in an intervention modifies their behaviour from what it would have been without the knowledge. In this study, patients in the intervention group will receive more attention than patients in the usual care group, which might lead to an overestimation of the effect of the intervention program. Although the Hawthorne effect cannot be prevented completely, the control group in this RCT continues to receive usual care. Therefore, they will not receive no attention at all.

A strength of this study is that, in contrast to previous studies, the intervention is executed at the workplace. As described by the ICF model, environmental factors will be involved in order to maintain and improve work productivity for workers with RA. Involving the supervisor in the intervention and adapting solutions for obstacles for functioning at work to the specific needs of patient and supervisor, is a substantial difference when compared to previous initiatives for workers with RA. Another strength of this study is that it builds on positive experiences of previous successful studies in other settings and populations [14,48].

### Impact of the results

Support for workers with RA to improve work productivity and maintain paid work is important for the individual workers, employees, and society. The previous study of Lambeek et al. (2010) showed a major improvement on return to work for workers with chronic low back pain who are completely or partially sick listed, by an intervention program consisting of integrated care, a participatory workplace intervention and graded activity. The solutions negotiated during the participatory workplace intervention were highly implemented. As described before, the current study is based on the intervention used in the study of Lambeek et al. (2010). High implementation of solutions negotiated during the participatory workplace intervention might lead to a substantial improvement in the work productivity of workers with RA, and the chances of implementation in usual care will be higher. When cost effective, it will be worth considering the integrated care intervention for usual care for workers with RA who come across difficulties at work.

Results are expected in 2015.

## Competing interests

The authors declare that they have no competing interests.

## Authors’ contributions

All authors contributed to the design of the article and the writing of this paper. They have all approved the final manuscript. MV is de principle researcher and is responsible for the data collection. CRLB coordinates the study. CRLB, RS, DS, AEV and JRA supervise the study.

## Pre-publication history

The pre-publication history for this paper can be accessed here:

http://www.biomedcentral.com/1471-2458/12/496/prepub
